# Comprehensive full-vessel segmentation and volumetric plaque quantification for intracoronary optical coherence tomography using deep learning

**DOI:** 10.1093/ehjdh/ztaf021

**Published:** 2025-03-15

**Authors:** Rick H J A Volleberg, Ruben G A van der Waerden, Thijs J Luttikholt, Joske L van der Zande, Pierandrea Cancian, Xiaojin Gu, Jan-Quinten Mol, Silvan Quax, Mathias Prokop, Clara I Sánchez, Bram van Ginneken, Ivana Išgum, Jos Thannhauser, Simone Saitta, Kensuke Nishimiya, Tomasz Roleder, Niels van Royen

**Affiliations:** Department of Cardiology, Radboud University Medical Center, PO Box 9101, 6500HB Nijmegen, The Netherlands; Department of Cardiology, Radboud University Medical Center, PO Box 9101, 6500HB Nijmegen, The Netherlands; Diagnostic Image Analysis Group, Radboud University Medical Center, Nijmegen, The Netherlands; Department of Cardiology, Radboud University Medical Center, PO Box 9101, 6500HB Nijmegen, The Netherlands; Diagnostic Image Analysis Group, Radboud University Medical Center, Nijmegen, The Netherlands; Department of Cardiology, Radboud University Medical Center, PO Box 9101, 6500HB Nijmegen, The Netherlands; Diagnostic Image Analysis Group, Radboud University Medical Center, Nijmegen, The Netherlands; Department of Biomedical Engineering and Physics, Amsterdam University Medical Center, Amsterdam, The Netherlands; Quantitative Healthcare Analysis Group, Informatics Institute, University of Amsterdam, Amsterdam, The Netherlands; Department of Biomedical Engineering and Physics, Amsterdam University Medical Center, Amsterdam, The Netherlands; Quantitative Healthcare Analysis Group, Informatics Institute, University of Amsterdam, Amsterdam, The Netherlands; Department of Cardiology, Radboud University Medical Center, PO Box 9101, 6500HB Nijmegen, The Netherlands; Diagnostic Image Analysis Group, Radboud University Medical Center, Nijmegen, The Netherlands; Department of Medical Imaging, Radboud University Medical Center, Nijmegen, The Netherlands; Department of Biomedical Engineering and Physics, Amsterdam University Medical Center, Amsterdam, The Netherlands; Quantitative Healthcare Analysis Group, Informatics Institute, University of Amsterdam, Amsterdam, The Netherlands; Diagnostic Image Analysis Group, Radboud University Medical Center, Nijmegen, The Netherlands; Department of Biomedical Engineering and Physics, Amsterdam University Medical Center, Amsterdam, The Netherlands; Quantitative Healthcare Analysis Group, Informatics Institute, University of Amsterdam, Amsterdam, The Netherlands; Department of Radiology and Nuclear Medicine, Amsterdam University Medical Center, Amsterdam, The Netherlands; Department of Cardiology, Radboud University Medical Center, PO Box 9101, 6500HB Nijmegen, The Netherlands; Diagnostic Image Analysis Group, Radboud University Medical Center, Nijmegen, The Netherlands; Department of Biomedical Engineering and Physics, Amsterdam University Medical Center, Amsterdam, The Netherlands; Quantitative Healthcare Analysis Group, Informatics Institute, University of Amsterdam, Amsterdam, The Netherlands; Department of Cardiovascular Medicine, Tohoku University Graduate School of Medicine, Sendai, Japan; Faculty of Medicine, Wrocław University of Science and Technology, Wrocław, Poland; Department of Cardiology, Radboud University Medical Center, PO Box 9101, 6500HB Nijmegen, The Netherlands

**Keywords:** Optical coherence tomography, Intracoronary, Artificial intelligence, Deep learning, Segmentation, Atherosclerotic plaque

## Abstract

**Aims:**

Intracoronary optical coherence tomography (OCT) provides detailed information on coronary lesions, but interpretation of OCT images is time-consuming and subject to interobserver variability. The aim of this study was to develop and validate a deep learning-based multiclass semantic segmentation algorithm for OCT (OCT-AID).

**Methods and results:**

A reference standard was obtained through manual multiclass annotation (guidewire artefact, lumen, side branch, intima, media, lipid plaque, calcified plaque, thrombus, plaque rupture, and background) of OCT images from a representative subset of pullbacks from the PECTUS-obs study. Pullbacks were randomly divided into a training and internal test set. An additional independent dataset was used for external testing. In total, 2808 frames were used for training and 218 for internal testing. The external test set comprised 392 frames. On the internal test set, the mean Dice score across nine classes was 0.659 overall and 0.757 on the true-positive frames, ranging from 0.281 to 0.989 per class. Substantial to almost perfect agreement was achieved for frame-wise identification of both lipid (κ=0.817, 95% CI 0.743–0.891) and calcified plaques (κ=0.795, 95% CI 0.703–0.887). For plaque quantification (e.g. lipid arc, calcium thickness), intraclass correlations of 0.664–0.884 were achieved. In the external test set, κ-values for lipid and calcified plaques were 0.720 (95% CI 0.640–0.800) and 0.851 (95% CI 0.794–0.908), respectively.

**Conclusion:**

The developed multiclass semantic segmentation method for intracoronary OCT images demonstrated promising capabilities for various classes, while having included difficult frames, such as those containing artefacts or destabilized plaques. This algorithm is an important step towards comprehensive and standardized OCT image interpretation.

## Graphical Abstract

**Graphical Abstract:**
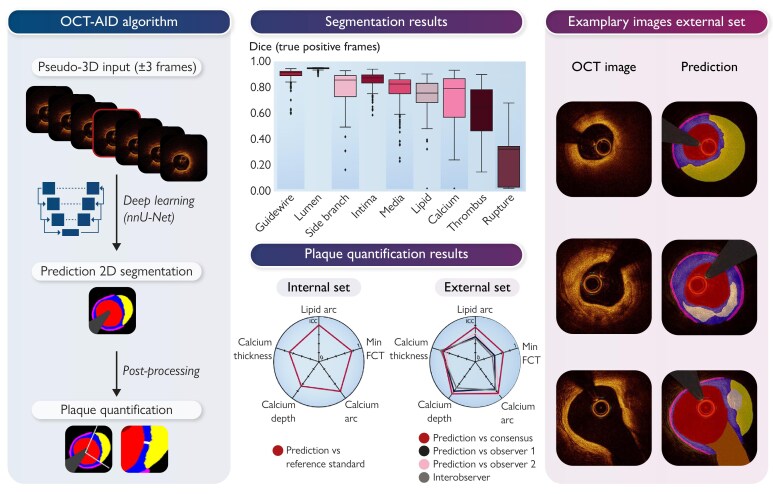
FCT, fibrous cap thickness; ICC, intraclass correlation; nnU-Net, No-new-Net; OCT, optical coherence tomography

## Introduction

Intracoronary optical coherence tomography (OCT) has distinct advantages compared with conventional coronary angiography, among which are its exceptionally high spatial resolution and the ability to visualize vessel wall morphology. Routine use of intracoronary imaging to optimize percutaneous coronary revascularization improves clinical outcome,^1^ and is now supported with a class I recommendation for guidance of complex lesion revascularization.^2^ Additionally, OCT can be a valuable tool to identify culprit lesions as well as high-risk plaques, lipidic plaques at increased risk of causing future cardiovascular events.^3,4^ Nevertheless, the clinical adoption of intracoronary imaging remains low.^5^ Limiting factors include unfamiliarity with OCT images, a prolonged procedure duration due to time-consuming image interpretation, and substantial interobserver variability.^6–8^

Automated OCT image interpretation through the application of deep learning (DL) can help overcome these limitations, and the high-resolution images obtained using OCT are particularly amenable to automated analyses. Indeed, commercially available tools already allow automated identification of lumen and vessel contours, calcified plaques, and stent struts, while algorithms for lumen and vessel contours are only now becoming available for intravascular ultrasound. Several other algorithms for OCT targeting these and other structures have been proposed over the past decades. In a recent systematic review from our group, we identified 91 articles on this topic. While the overall performance for the evaluation of the lumen and stent struts was very high, performance metrics for the identification and quantification of plaque types were generally lower. Furthermore, proposed algorithms predominantly focus on classification (e.g. classifying frames as plaque-containing or non-plaque-containing) or segmentation of a limited number of classes, and are not developed for complete OCT image interpretation. Importantly, external validation was only performed in 1 out of 8 studies.^9^ We therefore developed and validated a fully automatic, multiclass, DL-based algorithm for semantic segmentation of intracoronary OCT images to enable standardized image interpretation and reduce interobserver variability.

## Methods

### Study population and datasets

**Figure 1:**
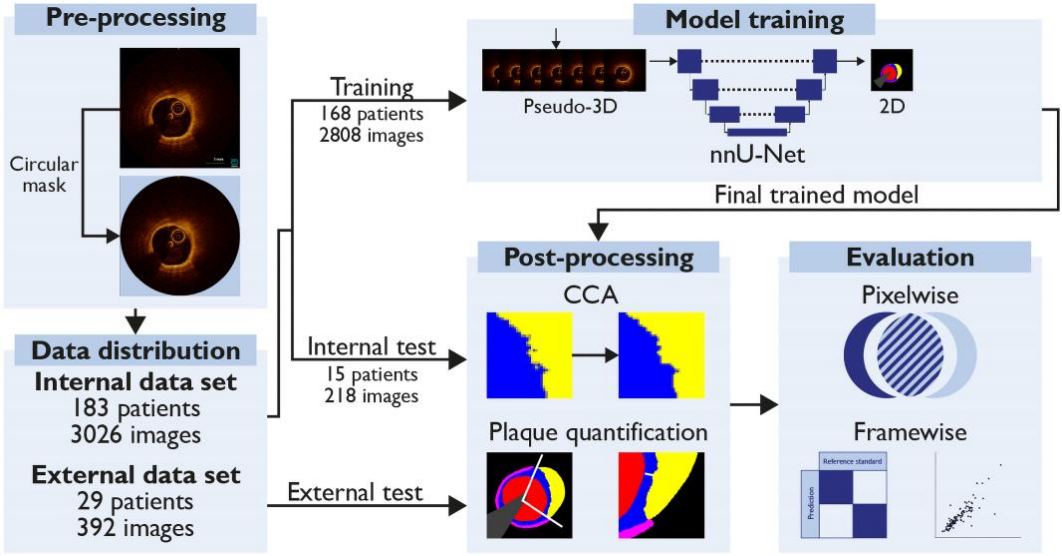
Study flow and OCT-AID pipeline. Study and algorithm flow for model training and evaluation. CCA, connected component analysis; nnU-Net no-new-Net.

This study is reported in accordance with the CLAIM reporting guidelines (see Supplementary material online, Method  *S1*). The internal dataset comprised a random subset of patients from the multinational, prospective PECTUS-obs study.^4,10^ In PECTUS-obs, 438 patients underwent OCT of all intermediate, non-flow limiting, non-culprit lesions after treatment of the infarct-related artery for ST-segment elevation or non-ST-segment elevation myocardial infarction between December 2018 and September 2020. Inclusion and exclusion criteria are provided in Supplementary material online, Method  *S2*. The study was conducted in accordance with the Declaration of Helsinki and its later amendments and was approved by the local medical-ethics committee or institutional review board of all participating centres. All patients provided informed consent prior to inclusion. The internal dataset was used for model development, including training and internal testing (*Figure 1*). Details on data splits are presented in Supplementary material online, Method  *S3*.

An independent external cohort of patients originating from a Core Lab registry (Real Solution Sp. Z o.o., Poland) was used for external testing. All patients underwent clinically indicated OCT, and inclusion was not limited to non-flow limiting lesions.

### Data acquisition and handling

All pullbacks were acquired using the Dragonfly Optis imaging catheter (Abbott Vascular, USA), predominantly with a pullback speed of 18 mm/s (54 mm) and 540 frames per pullback. OCT pullbacks were exported as DICOM files. Pullbacks were not used if an independent OCT core laboratory deemed the pullback of insufficient quality for further analysis. Additionally, frames without intima visibility due to significant artefacts (e.g. substantial blood artefacts) and frames with stent struts were excluded. Frames with destabilized plaques (i.e. thrombus or plaque rupture) or with artefacts that did not interfere with the interpretation were not excluded.

### Reference standard

The reference standard for model training and internal testing was defined by trained personnel with 1–4 years of experience in OCT image interpretation in accordance with contemporary guidelines.^3^ Supervision was provided by an interventional cardiologist with >10 years of experience with OCT. Pixel-wise annotation using ITK-SNAP 4.0^11^ was performed on every 40th frame of the pullback and on frames in-between in case typical plaque examples or examples with rare features (e.g. plaque rupture) were present. A total of ten labels were used for pixel-wise annotation: guidewire artefact, lumen, side branch, intima, media, lipid plaque, calcified plaque, thrombus, plaque rupture, and background. The abluminal border of lipid plaques was estimated visually based on the visible parts of the tunica media. Weekly meetings were held to discuss challenging frames and reach consensus where needed.

### Model architecture, training, and post-processing

We propose OCT-AID, which is based on the no-new-Net (nnU-Net),^12^ for multiclass semantic segmentation, which refers to the task of pixel-wise labelling the images. The nnU-Net is a state-of-the art, flexible framework for medical image segmentation, automatically adjusting its configuration to suit the given task and dataset. During training, the nnU-Net learns to assign each pixel in the input image to one of the predefined classes, effectively delineating different structures or regions within the image. To incorporate spatial information available within a pullback and to mimic expert visual analysis, the DL model was fed with seven consecutive cross-sectional frames stacked along the channel dimension (pseudo-3D). Preliminary results demonstrated better prediction results using seven frames as input compared with one, three, or five frames. The output was a 2D semantic segmentation of the central frame. During model testing, segmentation predictions were post-processed using connected component analysis to remove small, isolated predictions for all classes but plaque rupture and thrombus. Model architecture, training parameters and post-processing are detailed in Supplementary material online, Methods  *S3* and *S4*. The algorithm pipeline is presented in *Figure 1*.

### Plaque quantification

**Figure 2:**
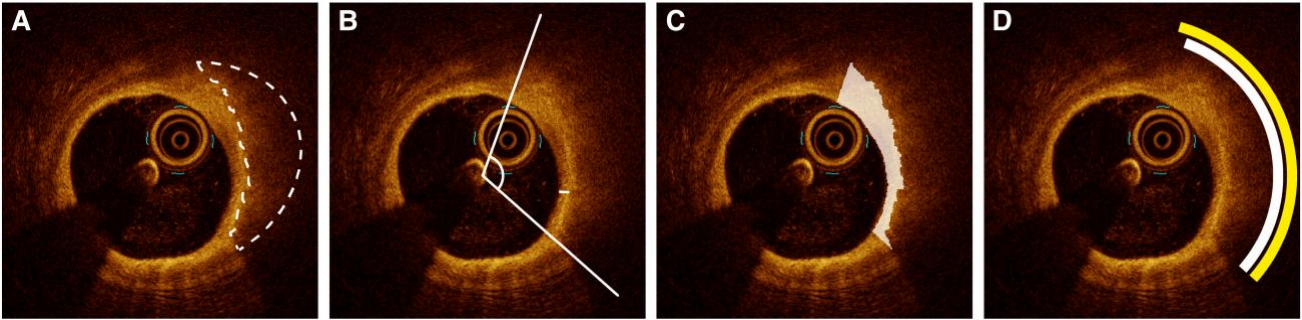
Lipid plaque quantification. Example of a lipid plaque delineated with white dashed line (*A*) and corresponding quantification measures, including the lipid arc and minimum fibrous cap thickness (*B*), fibrous cap area (*C*), and lipid arc correspondence score (*D*). In this particular example, the reference standard lipid arc was 112° (white arc) and the predicted lipid arc was 118° (yellow arc) with a correspondence of 112°, resulting in a correspondence score of 0.974.

Lipid and calcified plaques were quantified from the segmentation output (*Figure 2*). Arcs were calculated by evaluating the presence of each plaque type along ray casts as assessed from the centroid of the lumen. For plaques visible on both sides of the guidewire artefact, the arc was measured continuously across the guidewire artefact. The minimum fibrous cap thickness (FCT) and calcium depth were calculated as the minimum difference between lumen and lipid or calcium, respectively. The fibrous cap area was calculated as the total area labelled as intima within the lipid arc, and the fibrous cap ratio was defined as the fibrous cap area divided by the lipid arc. The calcium thickness was calculated as the maximum difference between the adluminal and abluminal border of a calcified plaque measured from the centroid of the lumen. A lipid-rich plaque was defined as a lipid plaque with an arc ≥90°.

### Internal testing

Pixel-wise segmentation performance was evaluated using the Dice similarity coefficient on the held-out internal test set. In addition, the DL model segmentation predictions were evaluated on a frame basis for their ability to identify each class in a binary fashion (i.e. absence or presence) and for plaque quantification compared with manual measurements. Cumulative lipid arcs, calcium arcs and fibrous cap areas were used when multiple non-overlapping areas of the same plaque type were present in a single frame. To further evaluate the correspondence in arc locations, a lipid and calcium arc correspondence score was calculated. This metric was defined as two times the correspondence in the lipid/calcium arcs (°) of the reference standard and prediction divided by the sum of the lipid/calcium arcs (°) of the reference standard and the prediction (*Figure 2D*).

### External testing

For external testing, OCT-AID was similarly evaluated compared with manual assessment of two independent interventional cardiologists (KN and TR) with large experience in OCT studies from different institutes. The independent assessors were blinded to the model predictions. Performance was evaluated on all frames in which both observers confirmed the presence or absence of the class evaluated. For continuous variables, the mean of the two independent assessors was used as reference. The Dice scores, arc correspondence scores, fibrous cap area and fibrous cap ratio were not evaluated in the external test set as this requires pixel-wise labelling. Performance of the algorithm was also evaluated on the complete external test set with direct comparison to one of the observers while the other observer served as reference, to allow a complete evaluation of the model performance on the complete dataset. Interobserver variability was evaluated by comparing the individual interpretations of the observers.

### Statistical analysis

Categorical baseline variables are expressed as numbers (percentages) and continuous variables are presented as means ± standard deviation. The χ^2^ tests and independent Student’s *t*-tests were used to evaluate categorical and continuous variables, respectively. Missing data were not imputed. Interobserver variability was assessed using Cohen’s kappa^13^ for frame-wise identification of each class, and intraclass correlation (ICC) for absolute agreement in a two-way random model^14^ for evaluation of quantification measures. Likewise, agreement between reference standard and predicted frame-wise class identification was evaluated using conventional performance metrics and Cohen’s kappa. Dice similarity coefficients for semantic segmentation are reported on all true-positive, false-positive, or false-negative frames as well as on true-positive frames only. Quantification measures were evaluated on true-positive frames only and measurements were presented in scatterplots and evaluated using ICC for absolute agreement in a two-way random model. Bias was assessed using Bland–Altman plots. 95%-Confidence intervals (CI) were calculated for all metrics. A two-sided *P*-value <0.05 was considered statistically significant. Statistical analyses were performed using SPSS Statistics version 27 (IBM Corp., USA) and RStudio version 2023.09.1 (PBC, USA).

## Results

### Study population

Among 438 patients included in PECTUS-obs, 183 were included in the present study with a total of 207 pullbacks and 3026 frames. These 183 patients were randomly included from the complete dataset of patients with at least one analysable OCT. One hundred sixty-eight patients (192 pullbacks and 2808 frames) were randomly assigned to the training set and 15 patients (15 pullbacks, 218 frames) to the internal test set (*Figure 1*). Baseline characteristics were well balanced between sets (*Table 1*). The frame-wise prevalence of each class is presented in Supplementary material online, *Table S1*.

**Table 1:** Patient-level baseline characteristics of included sets

Variable	Overall (*n* = 183)	Training (*n* = 168)	Test (*n* = 15)	*P*-value
Age, years	64 ± 10	64 ± 10	67 ± 10	0.354
Female sex	32 (17.5%)	29 (17.3%)	3 (20.0%)	0.729
Hypertension	87 (47.5%)	80 (47.6%)	7 (46.7%)	0.944
Diabetes	33 (18.0%)	29 (17.3%)	4 (26.7%)	0.480
Previous myocardial infarction	29 (15.8%)	27 (16.1%)	2 (13.3%)	1.000
Clinical scenario at presentation				
STEMI	74 (40.4%)	70 (41.7%)	4 (26.7%)	0.257
NSTEMI	109 (59.6%)	98 (58.3%)	11 (73.3%)	0.257

NSTEMI, non-ST-segment elevation myocardial infarction; STEMI, ST-segment elevation myocardial infarction.

### Semantic segmentation

**Figure 3:**
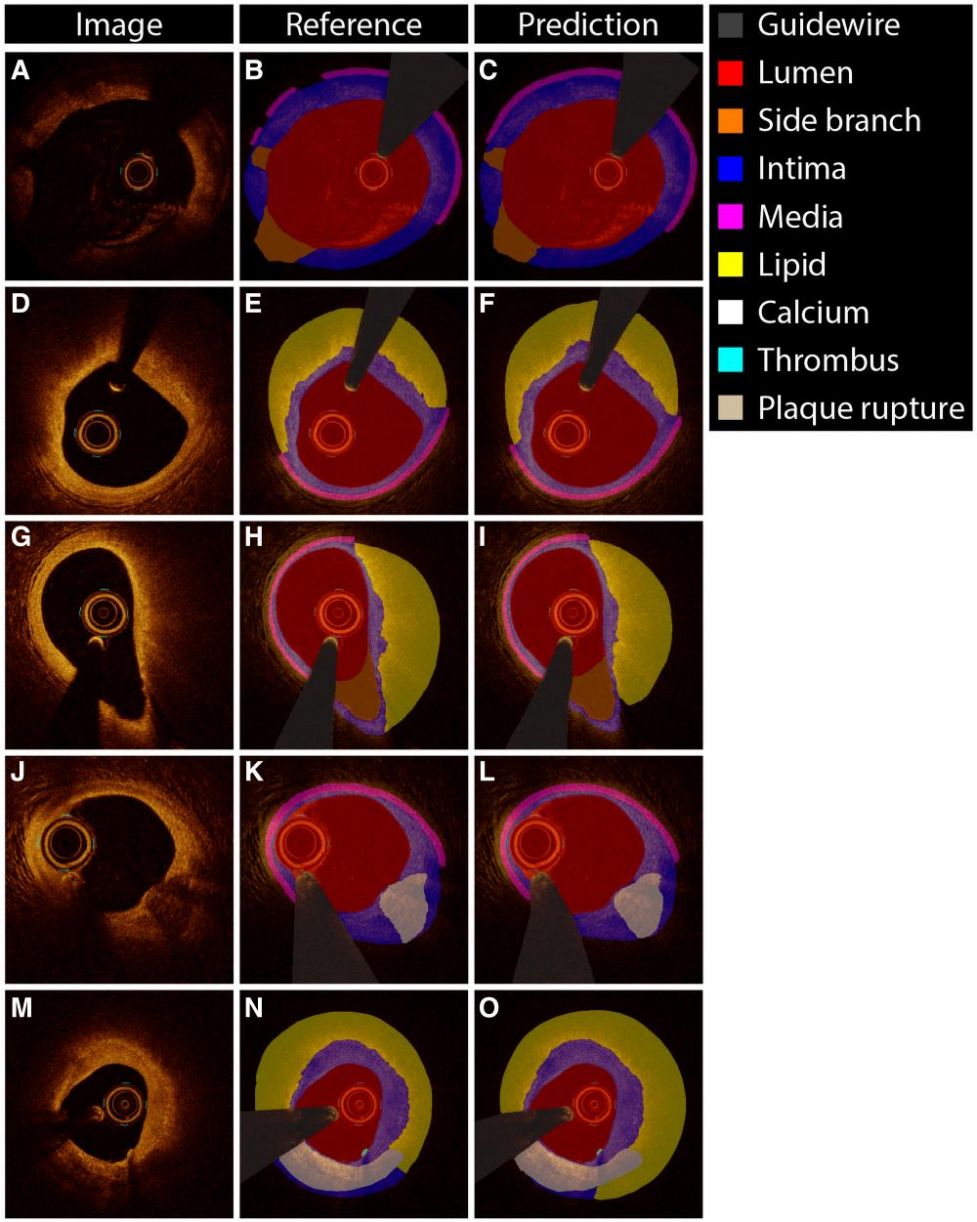
Multiclass semantic segmentation examples. Examples of the cross-sectional optical coherence tomography image (left column) reference standard (middle column) and predictions by OCT-AID (right column) in the held-out test set. (*A*–*C*) Frame affected by blood artefacts. (*D*–*F*) Lipid plaque. (*G*–*I*) Lipid plaque with a side branch. (*J*–*L*) Calcified plaque. (*M*–*O*) Combined lipid and calcified plaque and a small thrombus.

**Figure 4:**
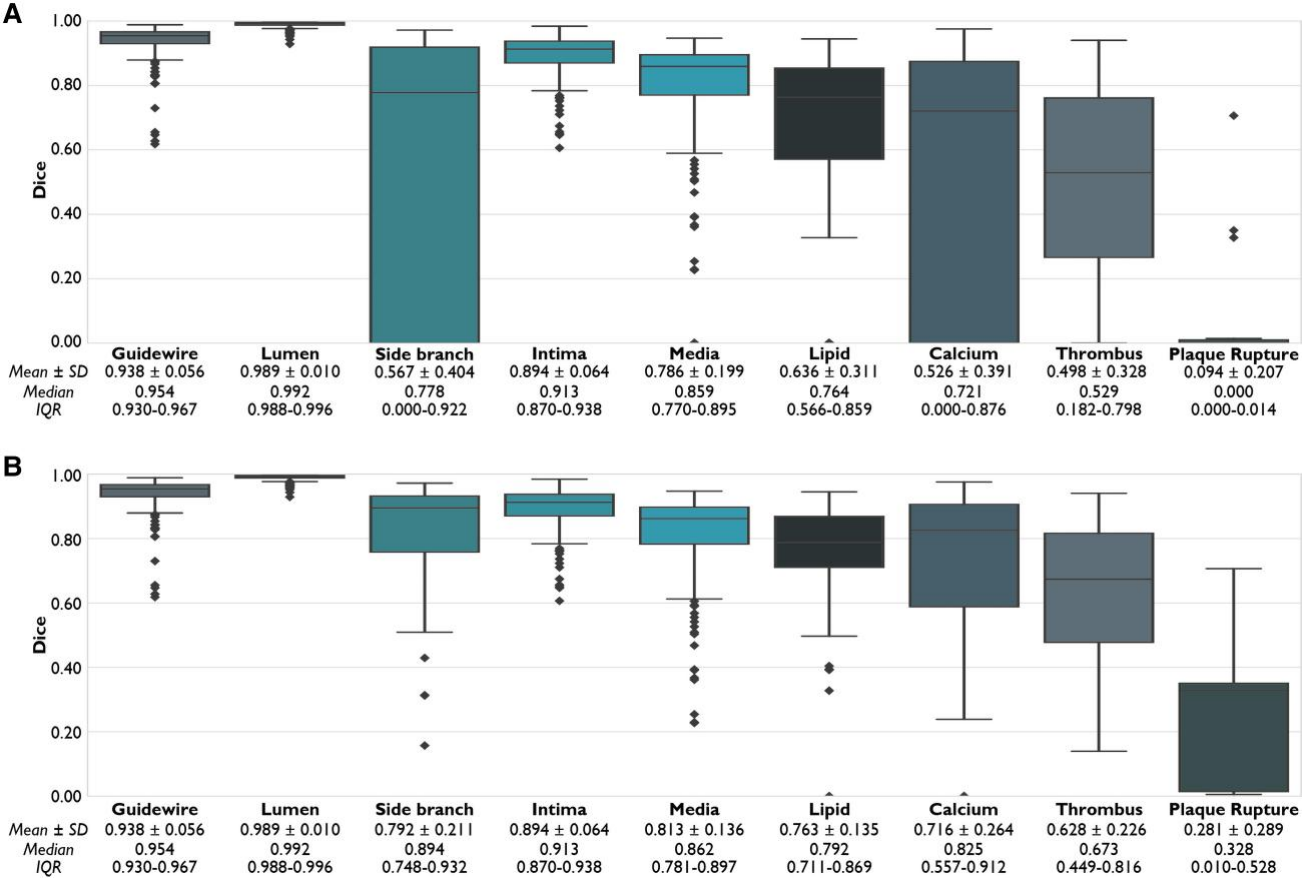
Per-frame dice score. Boxplots for the per-frame Dice score per class across all true-positive, false-positive, and false-negative frames (*A*) and across true-positive frames only (*B*). A score of 1 indicates perfect overlap and a score of 0 represent no overlap. False positive and false negative frames are awarded a score of 0. IQR, inter-quartile range; SD standard deviation.

*Figure 3* provides examples of the performance of OCT-AID for multiclass semantic segmentation. On the internal test set, the mean per class Dice score was 0.659 overall (*Figure 4A*). The mean Dice score for the lumen, intima, and media were 0.989 ± 0.010, 0.894 ± 0.064, and 0.786 ± 0.199, respectively. The mean Dice scores for lipid and calcified plaques were 0.636 ± 0.311 and 0.526 ± 0.391, respectively. The mean Dice scores obtained in the internal test set were comparable to the respective validation folds (see Supplementary material online, Method  *S2*). Among true-positive frames only, the mean per class Dice score was 0.757. Dice scores for lipid plaques, calcified plaques, side branch, thrombus, and plaque rupture were 0.763 ± 0.135, 0.716 ± 0.264, 0.792 ± 0.211, 0.628 ± 0.226, and 0.281 ± 0.289, respectively (*Figure 4B*).

### Frame-wise identification

In the internal test set, substantial to almost perfect agreement was achieved for frame-wise identification of lipid plaques (κ=0.817, 95% CI 0.743–0.891), lipid-rich plaque (κ=0.843, 95% CI 0.769–0.917), calcified plaques (κ=0.795, 95% CI 0.703–0.887), side branches (κ=0.736, 95% CI 0.624–0.848) and for thrombus (κ=0.871, 95% CI 0.759–0.983), and fair to substantial for plaque rupture (κ=0.480, 95% CI 0.210–0.750). Performance metrics and confusion matrices for each class are presented in *Table 2* and Supplementary material online, *Tables S2*–S7.

**Table 2:** Frame-wise performance metrics for identification and quantification

Class	Prevalence	TP frames	Accuracy (%)	Sensitivity (%)	Specificity (%)	PPV (%)	NPV (%)	Kappa/ICC^a^
Lipid plaque	102 (46.8%)	100 (98.0%)	90.8 (86.2–94.3)	98.0 (93.1–99.8)	84.5 (76.6–90.5)	84.7 (77.0–90.7)	98.0 (93.0–99.8)	0.817 (0.743–0.891)
Arc	–	–	–	–	–	–	–	0.814 (0.714–0.877)
Minimum FCT	–	–	–	–	–	–	–	0.766 (0.671–0.836)
FC area	–	–	–	–	–	–	–	0.768 (0.559–0.867)
FC ratio	–	–	–	–	–	–	–	0.884 (0.774–0.934)
LRP^b^	76 (34.9%)	73 (96.1%)	92.7 (88.4–95.7)	96.1 (88.9–99.2)	90.8 (84.9–95.0)	84.9 (75.5–91.7)	97.7 (93.5–99.5)	0.843 (0.769–0.917)
Calcified plaque	53 (24.3%)	47 (88.7%)	92.2 (87.8–95.4)	88.7 (77.0–95.7)	93.3 (88.4–96.6)	81.0 (68.6–90.1)	96.3 (92.0–98.6)	0.795 (0.703–0.887)
Arc	–	–	–	–	–	–	–	0.808 (0.444–0.917)
Depth	–	–	–	–	–	–	–	0.664 (0.455–0.801)
Thickness	–	–	–	–	–	–	–	0.690 (0.502–0.815)
Side branch	46 (21.1%)	36 (78.3%)	91.3 (86.7–94.7)	78.3 (63.6–89.1)	94.8 (90.3–97.6)	80.0 (65.4–90.4)	94.2 (89.6–97.2)	0.736 (0.624–0.848)
Thrombus	21 (9.6%)	19 (90.5%)	97.7 (94.7–99.3)	90.5 (69.6–98.8)	98.5 (95.6–99.7)	86.4 (65.1–97.1)	99.0 (96.4–99.9)	0.871 (0.759–0.983)
Plaque rupture	6 (2.8%)	5 (83.3%)	95.4 (91.7–97.8)	83.3 (35.9–99.6)	95.8 (92.1–98.0)	35.7 (12.8–64.9)	99.5 (97.3–100)	0.480 (0.210–0.750)

Metrics are reported as value (95% confidence interval).

FC, fibrous cap; FCT, fibrous cap thickness; ICC, intraclass correlation; LRP, lipid-rich plaque; NPV, negative predictive value; PPV, positive predictive value; TP, true-positive.

^a^Kappa is calculated for categorical variables (lipid plaque, calcified plaque, side branch, thrombus, and plaque rupture) and ICC is calculated for continuous variables (lipid arc, minimum FCT, FC area, FC ratio, calcium arc, calcium depth, and calcium thickness).

^b^Defined as a lipid plaque with an arc ≥90°.

### Lipid and calcified plaque quantification

In the internal test set, moderate to good reliability was achieved for the lipid arc measurements (ICC 0.814, 95% CI 0.714–0.877) with a mean difference of 16.3 ± 45.9° (see Supplementary material online, *Figure S1A* and *B*). The mean lipid arc correspondence score was 0.902 ± 0.120. For the minimum FCT, reliability was moderate to good (ICC 0.766, 95% CI 0.671–0.836). The mean difference was 6.8 ± 75.4 µm (see Supplementary material online, *Figure S1C* and *D*). The algorithm achieved moderate to good reliability for the fibrous cap area (ICC 0.768, 95% CI 0.559–0.867) and good to excellent reliability for the fibrous cap ratio (ICC 0.884, 95% CI 0.774–0.934) with a mean difference of 0.24 ± 0.42 mm^2^ and 0.0009 ± 0.0017 mm^2^/°, respectively (see Supplementary material online, *Figure S1E*-*H*).

Calcified plaque quantification and bias is reported in *Table 2* and Supplementary material online, *Figure S2*. The ICC for calcium arc, calcium depth, and calcium thickness were 0.808 (95% CI 0.444–0.917), 0.664 (95% CI 0.455–0.801), and 0.690 (95% CI 0.502–0.815), respectively. The mean calcium arc correspondence score was 0.829 ± 0.193.

### External evaluation

The external test set comprised 29 patients (29 pullbacks, 392 frames). *Table 3* provides an overview of the interobserver variability and the performance of OCT-AID compared with consensus in external testing. Supplementary material online, *Videos S1* and *S2* provide examples of the model interpretation including a challenging case with artefacts.

**Table 3:** External test set: interobserver variability and model performance across consensus frames

	Interobserver	External testing in consensus frames								
Class	Kappa/ICC^a^	Frames with consensus^b^	Prevalence	TP frames	Accuracy (%)	Sensitivity (%)	Specificity (%)	PPV (%)	NPV (%)	Kappa/ICC^a^
Lipid plaque	0.535 (0.451–0.619)	306 (78.1%)	104 (34.0%)	93 (89.4%)	86.9 (82.6–90.5)	89.4 (81.9–94.6)	85.6 (80.0–90.2)	76.2 (67.7–83.5)	94.0 (89.6–97.0)	0.720 (0.640–0.800)
Arc	0.516 (0.359–0.644)	–	–	–	–	–	–	–	–	0.761 (0.614–0.849)
Minimum FCT	0.392 (0.126–0.586)	–	–	–	–	–	–	–	–	0.649 (0.513–0.753)
LRP^c^	0.757 (0.665–0.849)	304 (77.6%)	64 (21.1%)	50 (78.1%)	92.1 (88.5–94.9)	78.1 (66.0–87.5)	95.8 (92.5–98.0)	83.3 (71.5–91.7)	94.3 (90.6–96.8)	0.757 (0.665–0.849)
Calcified plaque	0.905 (0.860–0.950)	376 (95.9%)	115 (30.6%)	105 (91.3%)	93.6 (90.7–95.9)	91.3 (84.6–95.8)	94.6 (91.2–97.0)	88.2 (81.0–93.4)	96.1 (93.0–98.1)	0.851 (0.794–0.908)
Arc	0.772 (0.566–0.869)	–	–	–	–	–	–	–	–	0.857 (0.623–0.931)
Depth	0.708 (0.569–0.802)	–	–	–	–	–	–	–	–	0.870 (0.815–0.910)
Thickness	0.803 (0.715–0.864)	–	–	–	–	–	–	–	–	0.756 (0.658–0.828)
Side branch	0.945 (0.906–0.984)	385 (98.2%)	77 (20.0%)	65 (84.4%)	94.0 (91.2–96.2)	84.4 (74.4–91.7)	96.4 (93.7–98.2)	85.5 (75.6–92.5)	96.1 (93.3–98.0)	0.812 (0.738–0.886)
Thrombus	0.560 (0.303–0.817)	383 (97.7%)	6 (1.6%)	4 (66.7%)	98.2 (96.3–99.3)	66.7 (22.3–95.7)	98.7 (96.9–99.6)	44.4 (13.7–78.8)	99.5 (98.1–99.9)	0.524 (0.214–0.834)
Plaque rupture	0.498 (−0.102–1.000)	390 (99.5%)	1 (0.3%)	0 (0%)	97.4 (95.3–98.8)	0.0 (0.0–97.5)	97.7 (95.7–98.9)	0.0 (0.0–33.6)	99.7 (98.5–100)	−0.005 (−0.013–0.003)

Metrics are reported as value (95% confidence interval).

FCT, fibrous cap thickness; ICC, intraclass correlation; LRP, lipid-rich plaque; NPV, negative predictive value; PPV, positive predictive value; TP, true positive.

^a^Kappa is calculated for categorical variables (lipid plaque, calcified plaque, side branch, thrombus, and plaque rupture) and ICC is calculated for continuous variables (lipid arc, minimum FCT, FC area, FC ratio, calcium arc, calcium depth, and calcium thickness).

^b^Refers to frames on which the independent observers agreed on the presence or absence of the evaluated class.

^c^Defined as a lipid plaque with an arc ≥90°.

**Figure 5:**
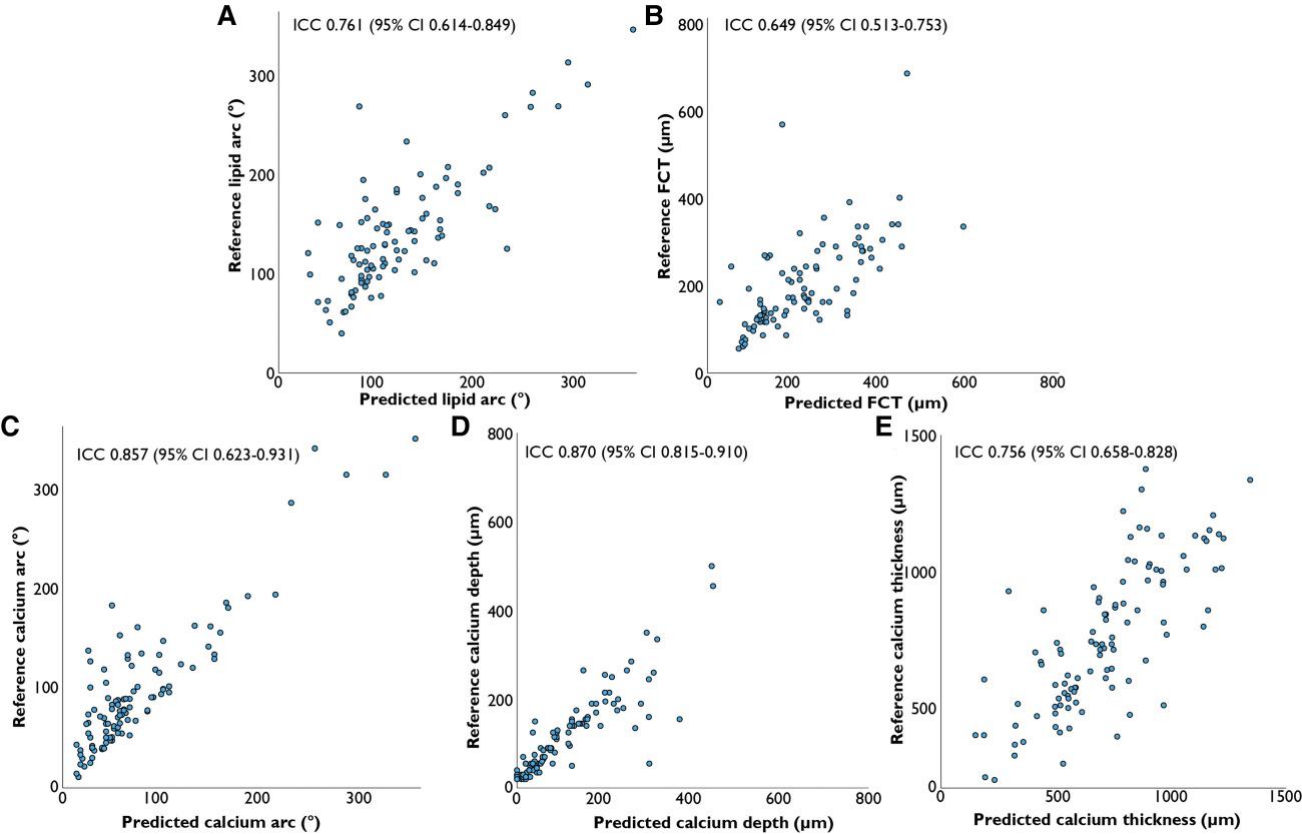
Correlation in plaque quantification in the external test set. Scatterplots for lipid arc (*A*), minimum fibrous cap thickness (*B*), calcium arc (*C*), calcium depth (*D*), and calcium thickness (*E*). CI, confidence interval; FC, fibrous cap; FCT, fibrous cap thickness, ICC, intraclass correlation.

The proposed algorithm had substantial agreement for frame-wise lipid plaque identification (κ=0.720, 95% CI 0.640–0.800) and substantial to almost perfect for lipid-rich plaques (κ=0.757, 95% CI 0.665–0.849). The ICCs for lipid arc (ICC 0.761, 95% CI 0.614–0.849) and minimum FCT (ICC 0.649, 95% CI 0.513–0.753) indicated moderate to good reliability (*Figure 5*). For calcified plaques, substantial to almost perfect agreement for frame-wise identification was achieved (κ=0.851, 95% CI 0.794–0.908). The ICCs for calcium arc, depth, and thickness indicate moderate to excellent (ICC 0.857, 95% CI 0.623–0.931), good to excellent (ICC 0.870, 95% CI 0.815–0.910), and moderate to good reliability (ICC 0.756, 95% CI 0.658–0.828), respectively (*Figure 5*). Bland–Altman plots are presented in Supplementary material online, *Figure S3*. Mean differences for the lipid arc, minimum FCT, calcium arc, calcium depth, and calcium thickness were −17.3 ± 40.3°, 18.8 ± 89.0 µm, −20.4 ± 29.3°, −3.8 ± 49.2 µm, and −38.9 ± 183.2 µm, respectively. Kappa values for side branch, thrombus, and plaque rupture were 0.812 (95% CI 0.738–0.886), 0.524 (95% CI 0.214–0.834), and −0.005 (95% CI −0.013–0.003), respectively. Evaluation metrics compared with one of the individual independent assessors as reference standard are presented in *Tables 4* and *5*.

**Table 4:** External test set: first observer as reference standard

Class	Prevalence	TP frames	Accuracy (%)	Sensitivity (%)	Specificity (%)	PPV (%)	NPV (%)	Kappa/ICC^a^
Second observer vs. first observer								
Lipid plaque	132 (33.7%)	104 (78.8%)	78.1 (73.6–82.1)	78.8 (70.8–85.4)	77.7 (72.1–82.6)	64.2 (56.3–71.6)	87.8 (82.9–91.8)	0.535 (0.451–0.619)
Arc	–	–	–	–	–	–	–	0.516 (0.359–0.644)
Minimum FCT	–	–	–	–	–	–	–	0.392 (0.126–0.586)
LRP^b^	91 (23.2%)	64 (70.3%)	77.6 (73.1–81.6)	70.3 (59.8–79.5)	79.7 (74.7–84.1)	51.2 (42.1–60.2)	89.9 (85.6–93.2)	0.443 (0.347–0.539)
Calcified plaque	127 (32.4%)	115 (90.6%)	95.9 (93.5–97.6)	90.6 (84.1–95.0)	98.5 (96.2–99.6)	96.6 (91.6–99.1)	95.6 (92.4–97.7)	0.905 (0.860–0.950)
Arc	–	–	–	–	–	–	–	0.772 (0.566–0.869)
Depth	–	–	–	–	–	–	–	0.708 (0.569–0.802)
Thickness	–	–	–	–	–	–	–	0.803 (0.715–0.864)
Side branch	83 (21.2%)	77 (92.8%)	98.2 (96.4–99.3)	92.8 (84.9–97.3)	99.7 (98.2–100)	98.7 (93.1–100)	98.1 (95.9–99.3)	0.945 (0.906–0.984)
Thrombus	9 (2.3%)	6 (66.7%)	97.7 (95.7–98.9)	66.7 (29.9–92.5)	98.4 (96.6–99.4)	50.0 (21.1–78.9)	99.2 (97.7–99.8)	0.560 (0.303–0.817)
Plaque rupture	1 (0.3%)	1 (100%)	99.5 (98.2–99.9)	100 (2.5–100)	99.5 (98.2–99.9)	33.3 (0.8–90.6)	100 (99.1–100)	0.498 (−0.102–1.000)
Model prediction vs. first observer								
Lipid plaque	132 (33.7%)	100 (75.8%)	71.9 (67.2–76.3)	75.8 (67.5–82.8)	70.0 (64.0–75.5)	56.2 (48.6–63.6)	85.0 (79.6–89.5)	0.421 (0.333–0.509)
Arc	–	–	–	–	–	–	–	0.552 (0.389–0.679)
Minimum FCT	–	–	–	–	–	–	–	0.447 (0.200–0.624)
LRP^b^	91 (23.2%)	57 (62.6%)	80.4 (76.1–84.2)	62.6 (51.9–72.6)	85.7 (81.2–89.5)	57.0 (46.7–66.9)	88.4 (84.1–91.8)	0.467 (0.365–0.569)
Calcified plaque	127 (32.4%)	107 (84.3%)	90.8 (87.5–93.5)	84.3 (76.7–90.1)	94.0 (90.4–96.5)	87.0 (79.7–92.4)	92.6 (88.8–95.4)	0.789 (0.724–0.854)
Arc	–	–	–	–	–	–	–	0.742 (0.358–0.875)
Depth	–	–	–	–	–	–	–	0.797 (0.715–0.857)
Thickness	–	–	–	–	–	–	–	0.753 (0.627–0.835)
Side branch	83 (21.2%)	69 (83.1%)	93.6 (90.7–95.8)	83.1 (73.3–90.5)	96.4 (93.7–98.2)	86.3 (76.7–92.9)	95.5 (92.6–97.5)	0.806 (0.733–0.879)
Thrombus	9 (2.3%)	4 (44.4%)	97.4 (95.4–98.8)	44.4 (13.7–78.8)	98.7 (97.0–99.6)	44.4 (13.7–78.8)	98.7 (97.0–99.6)	0.431 (0.141–0.721)
Plaque rupture	1 (0.3%)	0 (0%)	97.2 (95.0–98.6)	0.0 (0.0–97.5)	97.4 (95.3–98.8)	0.0 (0.0–30.8)	99.7 (98.6–100)	−0.005 (−0.013–0.003)

Metrics are reported as value (95% confidence interval).

FCT, fibrous cap thickness; ICC, intraclass correlation; LRP, lipid-rich plaque; NPV, negative predictive value; PPV, positive predictive value; TP, true-positive.

^a^Kappa is calculated for categorical variables (lipid plaque, calcified plaque, side branch, thrombus, and plaque rupture) and ICC is calculated for continuous variables (lipid arc, minimum FCT, FC area, FC ratio, calcium arc, calcium depth, and calcium thickness).

^b^Defined as a lipid plaque with an arc ≥90°.

**Table 5:** External test set: second observer as reference standard

Class	Prevalence	TP frames	Accuracy (%)	Sensitivity (%)	Specificity (%)	PPV (%)	NPV (%)	Kappa/ICC^a^
First observer vs. second observer								
Lipid plaque	162 (41.3%)	104 (64.2%)	78.1 (73.6–82.1)	64.2 (56.3–71.6)	87.8 (82.9–91.8)	78.8 (70.8–85.4)	77.7 (72.1–82.6)	0.535 (0.451–0.619)
Arc	–	–	–	–	–	–	–	0.516 (0.359–0.644)
Minimum FCT	–	–	–	–	–	–	–	0.392 (0.126–0.586)
LRP^b^	125 (31.9%)	64 (51.2%)	77.6 (73.1–81.6)	51.2 (42.1–60.2)	89.9 (85.6–93.2)	70.3 (59.8–79.5)	79.7 (74.7–84.1)	0.443 (0.347–0.539)
Calcified plaque	119 (30.4%)	115 (96.6%)	95.9 (93.5–97.6)	96.6 (91.6–99.1)	95.6 (92.4–97.7)	90.6 (84.1–95.0)	98.5 (96.2–99.6)	0.905 (0.860–0.950)
Arc	–	–	–	–	–	–	–	0.772 (0.566–0.869)
Depth	–	–	–	–	–	–	–	0.708 (0.569–0.802)
Thickness	–	–	–	–	–	–	–	0.803 (0.715–0.864)
Side branch	78 (19.9%)	77 (98.7%)	98.2 (96.4–99.3)	98.7 (93.1–100)	98.1 (95.9–99.3)	92.8 (84.9–97.3)	99.7 (98.2–100)	0.945 (0.906–0.984)
Thrombus	12 (3.1%)	6 (50.0%)	97.7 (95.7–98.9)	50.0 (21.1–78.9)	99.2 (97.7–99.8)	66.7 (29.9–92.5)	98.4 (96.6–99.4)	0.560 (0.303–0.817)
Plaque rupture	3 (0.8%)	1 (33.3%)	99.5 (98.2–99.9)	33.3 (0.8–90.6)	100.0 (99.1–100)	100.0 (2.5–100)	99.5 (98.2–99.9)	0.498 (−0.102–1.000)
Model prediction vs. second observer								
Lipid plaque	162 (41.3%)	142 (87.7%)	85.7 (81.9–89.0)	87.7 (81.6–92.3)	84.3 (79.0–88.8)	79.8 (73.1–85.4)	90.7 (85.9–94.2)	0.710 (0.639–0.781)
Arc	–	–	–	–	–	–	–	0.630 (0.466–0.742)
Minimum FCT	–	–	–	–	–	–	–	0.449 (0.309–0.571)
LRP^b^	125 (31.9)	83 (66.4%)	84.9 (81.0–88.3)	66.4 (57.4–74.6)	93.6 (90.0–96.2)	83.0 (74.2–89.8)	85.6 (81.1–89.4)	0.634 (0.550–0.718)
Calcified plaque	119 (30.4%)	107 (89.9%)	92.9 (89.8–95.2)	89.9 (83.0–94.7)	94.1 (90.7–96.6)	87.0 (79.7–92.4)	95.5 (92.3–97.7)	0.833 (0.774–0.892)
Arc	–	–	–	–	–	–	–	0.895 (0.840–0.930)
Depth	–	–	–	–	–	–	–	0.851 (0.784–0.898)
Thickness	–	–	–	–	–	–	–	0.682 (0.566–0.772)
Side branch	78 (19.9%)	65 (83.3%)	92.9 (89.8–95.2)	83.3 (73.2–90.8)	95.2 (92.2–97.3)	81.2 (71.0–89.1)	95.8 (93.0–97.8)	0.778 (0.700–0.856)
Thrombus	12 (3.1%)	4 (33.3%)	96.7 (94.4–98.2)	33.3 (9.9–65.1)	98.7 (97.0–99.6)	44.4 (13.7–78.8)	97.9 (95.9–99.1)	0.364 (0.095–0.633)
Plaque rupture	3 (0.8%)	1 (33.3%)	97.2 (95.0–98.6)	33.3 (0.8–90.6)	97.7 (95.7–98.9)	10.0 (0.3–44.5)	99.5 (98.1–99.9)	0.144 (−0.123–0.411)

Metrics are reported as value (95% confidence interval).

FCT, fibrous cap thickness; ICC, intraclass correlation; LRP, lipid-rich plaque; NPV, negative predictive value; PPV, positive predictive value; TP true-positive.

^a^Kappa is calculated for categorical variables (lipid plaque, calcified plaque, side branch, thrombus, plaque rupture) and ICC is calculated for continuous variables (lipid arc, minimum FCT, FC area, FC ratio, calcium arc, calcium depth, and calcium thickness).

^b^Defined as a lipid plaque with an arc ≥90°.

### Algorithm misidentification

**Figure 6:**
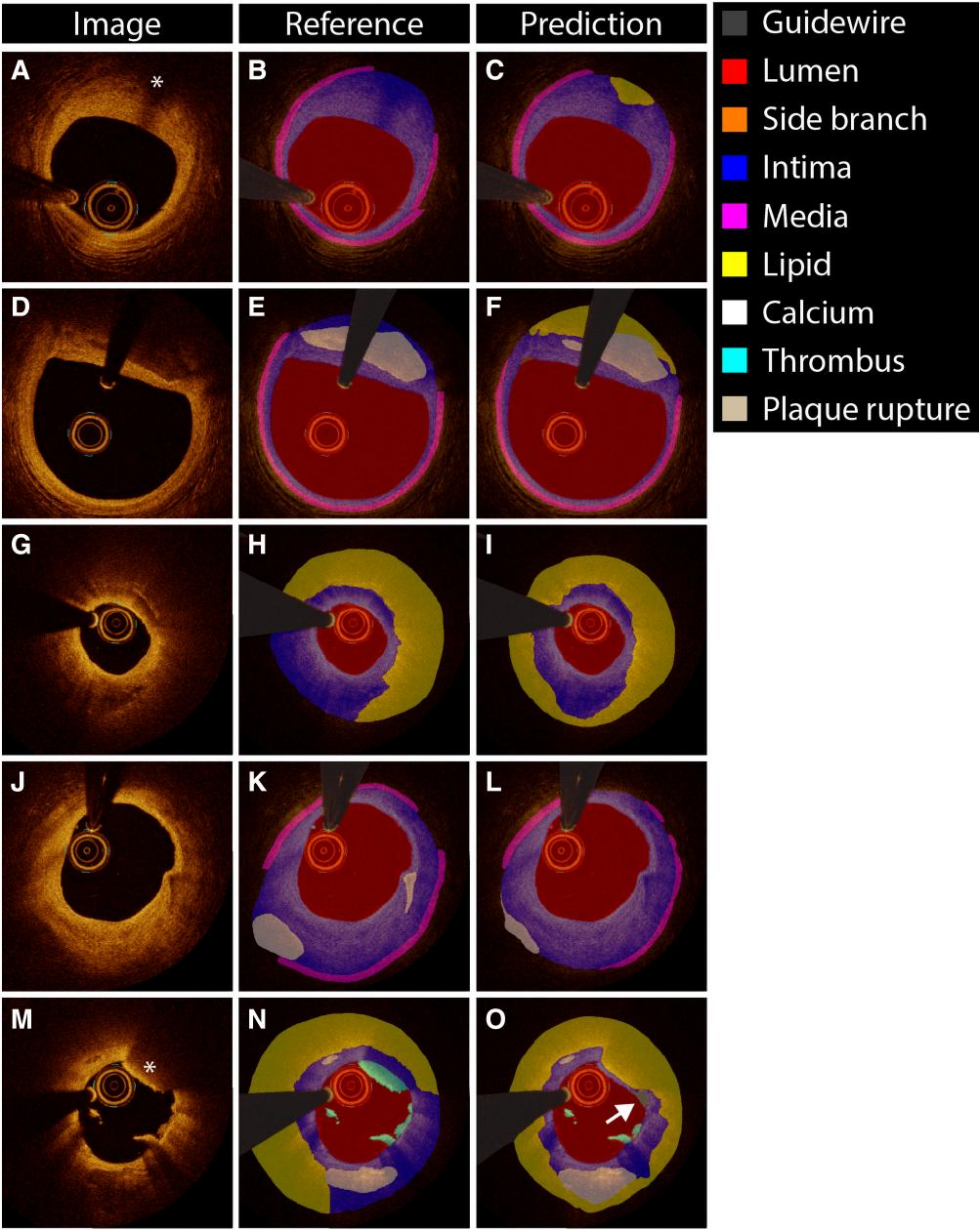
Algorithm misidentification. Examples of the cross-sectional optical coherence tomography image (left column) reference standard (middle column) and prediction by OCT-AID (right column) in the held-out test set. False positive predictions of lipid due to artefacts (*A*-*C*, asterisk in *A*) or at the abluminal side of a large calcified plaque (*D–F*). Overestimation of the lipid arc in presence of a large layered plaque/intraplaque hemorrhage (*G–I*). False negative prediction of a small calcification, resulting in a substantial underestimation of the calcium depth (*J–L*). False prediction of plaque rupture (arrow in *O*) and false negative prediction of thrombus at 2 O’ clock (asterisk in *M*) (*M–O*).

Exemplary images of disagreement between the reference standard and the prediction by OCT-AID are presented in *Figure 6*. Among false positive predictions of lipid, 38.9% (7/18) in the internal test set and 27.6% (8/29) in the external test set were predictions of lipid behind large calcifications. False positive lipid diagnoses were also found in presence of attenuating artefacts (22.2% in the internal test set and 13.8% in the external test set). Regarding the low positive predictive value for plaque rupture, the majority of false positive predictions were found in frames exhibiting thrombus without plaque rupture.

### Inference time

Inference was run on a NVIDIA GeForce RTX 3080 with 64 gigabytes of memory. Mean analysis time was 0.84 ± 0.03 s/frame, including 0.05 ± 0.02 s/frame for pre-processing, 0.73 ± 0.03 s/frame for inference, and 0.07 ± 0.01 s/frame for post-processing.

## Discussion

The proposed OCT-AID algorithm for multiclass semantic segmentation of intracoronary OCT images achieved promising performance for semantic segmentation and frame-wise identification of several plaque-related and non-plaque-related features. Besides conventional plaque quantification, for which the reliability was overall moderate to excellent, OCT-AID allows further volumetric plaque quantification. Importantly, these results were obtained while including difficult frames, such as those containing artefacts (*Figure 3A–C*; Supplementary material online, *Video S2*) or destabilized plaques (*Figure 5M–O*), which is in contrast to earlier works. With future improvements addressing its current challenges, including optimization of the algorithm for combined or destabilized plaques, and optimization of inference times, OCT-AID has the potential to allow accurate, standardized OCT image interpretation and reduce interobserver variability.

### Automated image interpretation

Time-consuming image analysis of intracoronary OCT images and substantial intraobserver and interobserver variability remain a clinical challenge.^6–8^ As a result, real-time evaluation during a clinical procedure is limited to scanning of pullbacks, and *post hoc* core lab analyses are generally limited to single frame evaluations at set intervals. To overcome this challenge, several AI-based approaches have been investigated previously. However, these were predominantly classification algorithms or segmentation models with a limited number of classes.^9^

Conversely, OCT-AID was trained for comprehensive image interpretation. The average Dice score of our approach was 0.659 across nine classes, including an excellent performance for semantic segmentation of the lumen, intima and media. For lipid and calcified plaques, Dice scores among true-positive frames were 0.763 ± 0.135 and 0.716 ± 0.264. However, expressing the model performance for semantic segmentation of lipid and thick calcified plaques in terms of Dice score may be inadequate, owing to the limited penetration depth of OCT. Indeed, the arc correspondence scores of 0.902 ± 0.120 and 0.829 ± 0.193 for lipid and calcified plaques confirm the ability of our algorithm to correctly localize these plaques. These results were obtained within <1 s/frame using hardware feasible for daily clinical care, which is considerably faster compared with manual labelling, which can take up to 30 min/frame but slightly slower than the median in a recent systematic review.^9^ However, our algorithm provides multiclass segmentation, while the referenced median value also comprises algorithms for single class segmentation. Nevertheless, higher-end hardware and model adaptions may further reduce inference times towards real-time full-vessel segmentation.

Only a limited number of other multiclass segmentation models have been proposed previously. Shibutani *et al*. developed a DL algorithm for multiclass (fibrocalcific plaque, pathological intimal thickening, lipid plaque, layered plaque, and background) semantic segmentation using 1103 OCT frames, co-registered with histopathology, as reference standard. In the held-out test set, the Jaccard index for lipid, calcified, and layered plaques were 0.45, 0.51, and 0.36, respectively.^15^ Chu *et al*. used a U-net for multiclass (fibrous plaque, lipid plaque, calcified plaque, cholesterol crystals, macrophages, microvessels, guidewire artefact, side branches, and internal elastic lamina) semantic segmentation and achieved a mean per class Dice score of 0.764. The Dice score for lipid and calcified plaques were 0.772 and 0.848 in internal testing and accuracy for region-wise identification of these plaques were 90.5% and 88.5% in external testing.^16^ Comparing those results to the present findings is however challenging for several reasons. First and foremost, it is generally unclear whether Dice scores in previous efforts were published on true-positive frames only or whether false positive and false negative frames were also included. Hence, we report our metrics in both scenarios to allow future comparison of our results to other algorithms. Second, challenging frames exhibiting plaque rupture, thrombus, dissections, and intramural hematoma were excluded by Chu *et al*. Third, expert analysts were asked to classify pre-delineated regions for external testing in the study by Chu *et al*., which may have biased their decision-making, compared with evaluation of a non-delineated frame as was performed in the present study.

The performance in the external test set appeared lower for some classes compared with the internal test set. We hypothesize that the difference in study population between the two sets had the most profound effect on this disparity. In particular, the internal test set comprised images of non-obstructive lesions performed for research purposes, while the external dataset contained OCT images from daily clinical care, which are more likely to be obstructive as OCT is predominantly performed for stent guidance and optimization. Therefore, future data enrichment for obstructive and potentially culprit lesions is warranted. Additionally, large scale external testing studies, including a web-based reader study, are needed and being planned to confirm the findings of the present study.

### Lipid plaque identification and quantification

Identification and quantification of lipid plaques is particularly challenging for humans, as highlighted by the substantial interobserver variability for these tasks in the present study and in literature.^7^ For automated identification of lipid plaques, the performances of our proposed methodology were at least on par with manual interobserver agreement by two expert readers. However, difficulties remain in specific situations, including presence of combined lipid and calcified (*Figure 6D–F*), layered (*Figure 6G–I*), or destabilized plaques (*Figure 6M–O*). Particularly, we observed that a substantial proportion of false positive predictions were related to lipid predictions behind large calcifications. From a clinical point of view, identification of such deep lipid plaques may have limited value, which may have led to a lower identification rate by the independent assessors. In contrast, semantic segmentation requires labelling of each pixel which may have led to the models’ overprediction of lipid in these deep-lying low intensity areas. However, judging by experience from newest generation OCT catheters with superior penetration depth, the algorithm may also be correct.^17^ Future histology-based evaluation studies of AI-algorithms may provide additional insights.

OCT-AID also allows quantification of lipid plaques including the fibrous cap. So far, identification of high-risk plaques predominantly relies on manual single-point measures of the FCT, which may only provide a part of the puzzle as it omits volumetric and spatial information.^18^ The earliest approaches for automated quantification of the fibrous cap were based on mathematical models related to changes in light intensity.^19–21^ DL has been tested to this regard more recently. An algorithm proposed by Lee *et al*. significantly underestimated the minimum FCT (0.121 ± 0.024 vs. 0.151 ± 0.035 mm).^22^ In another hybrid approach, using both DL and dynamic programming, the mean difference in the minimum FCT between two users was of 4.2 ± 14.6 µm after manual modification of the FCT in 5.5 ± 0.9% of frames.^23^ However, these values only reflect the interuser reproducibility, and a comparison between the algorithm-derived FCT with manual measurement is lacking. In a recent study specifically targeting segmentation of the fibrous cap, the R^2^ for mean FCT was 0.909 in a held-out test set comprising 1362 images from 74 pullbacks.^24^ However, the FCT was evaluated on A-lines which can result in an overestimation in cases with eccentric OCT catheter placement, and the minimum FCT was not evaluated.

Our approach demonstrates promising results for quantification of the fibrous cap, including area-based measures with moderate to excellent reliability for the fibrous cap ratio. These area-based measures can be transformed in volumetric measures that provide theoretical benefits compared with single-point measurement, so far not tested in studies. Indeed, an association between such volumetrics measures and clinical outcome is yet to be established and our area-based measures require external validation.

### Calcified plaque identification

OCT-AID achieved particularly high performance for the identification and quantification of calcified plaques. However, a small number of outliers negatively impacted the correlation coefficients (*Figure 6J–L*;  Supplementary material online, *Figure S2C*-*D*). Additionally, Bland-Altman analysis revealed a slight underestimation of the model compared with the reference standard for calcium arc and thickness.

The proposed algorithm may allow differentiating distinct calcification types based on their depth, arcs and volumes. Additionally, elaborate calcified plaque quantification can become helpful for downstream tasks such as the prediction of acute procedural success. To that regards, Gharaibeh *et al*. recently demonstrated that a machine learning algorithm with multiple lumen and calcification-related features, including calcification area and volume, outperformed the conventional calcium score for the prediction of stent expansion.^25^ These results indicate that additional predictive value can be obtained by incorporating more elaborate calcification-related variables. However, these cannot be obtained manually on-site, emphasizing the need for automated analyses.

### Thrombus and plaque rupture identification

In the present study, the model performance for the identification of thrombus and especially plaque rupture was substantially lower than for other classes. However, the low prevalence of these classes precluded a thorough evaluation. Nevertheless, we observed that OCT-AID had the tendency to predict presence of plaque rupture whenever there was thrombus predicted (*Figure 6M–O*), which requires further attention. However, our rationale to include these classes was to provide contextual information and to increase the generalizability of our algorithm by, in contrast to previous attempts, not excluding such frames. Separate dedicated algorithms targeting identification of such features will likely be more efficient and could subsequently be combined with a multiclass model.

## Limitations

This study has some limitations. First, only non-flow limiting non-culprit lesions were used for training and internal testing. However, the external test set comprised a diverse dataset of patients undergoing clinically indicated OCT. Second, the outer border of the lipid pool was estimated based on the visible parts of the media and it has not been correlated to intracoronary ultrasound. This inherent limitation of intracoronary OCT limits the evaluation of plaque burden. Third, although images with artefacts were included in the training and test sets, those with severe artefacts limiting accurate manual segmentation were excluded. Increasing the number of frames with moderate artefacts may enhance the generalizability. Fourth, the performance of OCT-AID for the segmentation and identification of plaque rupture and to a smaller degree thrombus was suboptimal and requires further attention and enrichment of the dataset for these classes. Last, computational constraints precluded incorporation of a large number of frames in the pseudo-3D approach or a full 3D approach.

## Conclusion

Our proposed OCT-AID algorithm for multiclass semantic segmentation of intracoronary OCT images demonstrated promising capabilities for the identification and quantification of various non-plaque-related and plaque-related features. Importantly, these results were obtained while including difficult frames, such as those containing artefacts or destabilized plaques, which is in contrast to earlier works. This algorithm has the potential to allow comprehensive and standardized OCT image interpretation.

## Supplementary Material

ztaf021_Supplementary_Data

## Data Availability

The data underlying this article are available from the corresponding author upon reasonable request. The code and annotated data underlying this manuscript cannot be made publicly available due to funding source restrictions.
