# Supplementary material for: Comprehensive full-vessel segmentation and volumetric plaque quantification for intracoronary optical coherence tomography using deep learning
Source: Eur Heart J Digit Health. 2025 Mar 15;6(3):404–16. doi: 10.1093/ehjdh/ztaf021 (PMC12088710; doi:10.1093/ehjdh/ztaf021)
Supplement: ztaf021_Supplementary_Data [file ztaf021_supplementary_data.zip › Supplementary_material_online.docx]

**Supplementary Appendix**

Content

[Supplementary Methods 2](#_Toc193805864)

[Method S1 CLAIM checklist 2](#_Toc193805865)

[Method S2 Inclusion and exclusion criteria of internal dataset 3](#_Toc193805866)

[Method S3 OCT-AID development 4](#_Toc193805867)

[Method S4 Mean overall Dice score without postprocessing in early stopping experiment 7](#_Toc193805868)

[Supplementary Results 8](#_Toc193805869)

[Table S1 Class distribution within internal test set 8](#_Toc193805870)

[Table S2 Confusion matrix for frame-wise lipid plaque identification 9](#_Toc193805871)

[Table S3 Confusion matrix for frame-wise lipid-rich plaque identification 9](#_Toc193805872)

[Table S4 Confusion matrix for frame-wise calcified plaque identification 10](#_Toc193805873)

[Table S5 Confusion matrix for frame-wise side branch identification 10](#_Toc193805874)

[Table S6 Confusion matrix for frame-wise thrombus identification 11](#_Toc193805875)

[Table S7 Confusion matrix for frame-wise plaque rupture identification 11](#_Toc193805876)

[Figure S1 Correlation and bias in lipid plaque quantification in the internal test set 12](#_Toc193805877)

[Figure S2 Correlation and bias in calcified plaque quantification in the internal test set 14](#_Toc193805878)

[Figure S3 Bias in plaque quantification in the external test set 15](#_Toc193805879)

[Supplementary References 16](#_Toc193805880)

# Supplementary Methods

## Method S1 CLAIM checklist

| Section/Topic | No. | Item | Page |
| --- | --- | --- | --- |
| Title/abstract |  |  |  |
|  | 1 | Identification as a study of AI methodology, specifying the category of technology used (e.g., deep learning) | 1 |
| Abstract |  |  |  |
|  | 2 | Summary of study design, methods, results and conclusions | 4-5 |
| Introduction |  |  |  |
|  | 3 | Scientific and/or clinical background, including the intended use and role of the AI approach | 9 |
|  | 4 | Study aims, objectives, and hypotheses | 9 |
| Methods |  |  |  |
| Study design | 5 | Prospective or retrospective design | 10 |
|  | 6 | Study goal | 9 |
| Data | 7 | Data sources | 10 |
|  | 8 | Inclusion and exclusion criteria | 10, A3 |
|  | 9 | Data pre-processing | A4 |
|  | 10 | Selection of data subsets | 10, A4 |
|  | 11 | De-identification methods | A4 |
|  | 12 | How missing data were handled | 13 |
|  | 13 | Image acquisition protocol | 10 |
| Reference Standard | 14 | Definition of method(s) used to obtain reference standard | 11-13 |
|  | 15 | Rationale for choosing the reference standard | 11-13 |
|  | 16 | Source of reference standard annotations | 11-13 |
|  | 17 | Annotation of test set | 11-13 |
|  | 18 | Measures of inter- and intra-rater variability of features described by the annotators | 13 |
| Data Partitions | 19 | How data were assigned to partitions | 10, A4 |
|  | 20 | Level at which partitions are disjoint | A4 |
| Testing Data | 21 | Intended sample size | NA |
| Model | 22 | Detailed description of model | 11, A4-5 |
|  | 23 | Software libraries, frameworks, and packages | NA |
|  | 24 | Initialization of model parameters | A5 |
| Training | 25 | Details of training approach | 11, A5 |
|  | 26 | Method for selecting the final model | A5, A7 |
|  | 27 | Ensembling techniques | A5 |
| Evaluation | 28 | Metrics of model performance | 12-13 |
|  | 29 | Statistical measures of significance and uncertainty | 13 |
|  | 30 | Robustness or sensitivity analysis | Not performed |
|  | 31 | Methods for explainability or interpretability | NA |
|  | 32 | Evaluation on internal data | 12 |
|  | 33 | Testing on external data | 12-13 |
|  | 34 | Clinical trial registration | NA |
| Results |  |  |  |
| Data | 35 | Numbers of patients or examinations included and excluded | 14, 15, F1 |
|  | 36 | Demographic and clinical characteristics of cases in each partition | 14 |
| Model performance | 37 | Performance metrics and measures of statistical uncertainty | 14-16 |
|  | 38 | Estimates of diagnostic performance and their precision | 14-16 |
|  | 39 | Failure analysis of incorrect results | 16 |
| Discussion |  |  |  |
|  | 40 | Study limitations | 21 |
|  | 41 | Implications for practice, including intended use and/or clinical role | 17 |
| Other information |  |  |  |
|  | 42 | Provide a reference to the full study protocol or to additional technical details | NA |
|  | 43 | Statement about the availability of software, trained model, and/or data | 2 |
|  | 44 | Sources of funding and other support; role of funders | 2-3 |

A Appendix; NA not applicable.

## Method S2 Inclusion and exclusion criteria of internal dataset

Patients were eligible for inclusion if they had at least one intermediate (visually estimated stenosis of 30-90%) non-culprit lesion that was fractional flow reserve negative (>0.80) and that was not a case of in-stent restenosis.

Exclusion criteria were <18 years of age, hemodynamic instability, previous coronary artery bypass grafting, anatomy of target lesion(s) unsuitable for optical coherence tomography (OCT) catheter crossing or imaging (i.e. aorta-ostial lesions, too small diameter segments, severe calcifications, chronic total occlusion, very distal lesions), pregnancy and an estimated life expectancy <3 years.

## Method S3 OCT-AID development

*Data handling of the internal dataset*

OCT images from the internal dataset were randomly divided on a patient-level into a training and test set using a stratified 9:1 ratio. This non-overlapping split maintained similar class distributions on a frame level between the two sets (Supplementary Table 1), with discrepancies kept below 10% per class. Image quality was not systematically evaluated and therefore not taken into account during data splitting.

The training set was in turn divided on a patient-level into five non-overlapping train/validation subsets, which were used for 5-fold cross validation as detailed in the model training section below. These subsets were similarly stratified to preserve equal class distributions. The internal test set was held-out during training of the model and was used for evaluation of the model performance after the final model was established.

*Data preprocessing*

Each OCT pullback was exported as an RGB image with an isotropic in-plane pixel size of 9.9 µm/pixel, cross-sectional dimensions of 704 x 704 pixels per frame and variable longitudinal extent (predominantly 54 mm). The field of view was set to 7 mm, and a circular mask with a radius of 352 was applied to delete watermarks. Image intensities were standardized using Z-score normalization, ensuring that each frame in the dataset had zero mean and unit variance. To leverage spatial context within a pullback and emulate expert image analysis, each frame was augmented with three upstream and three downstream adjacent frames, concatenated along the channel dimension. Therefore, the final input of the model was a 704 x 704 tensor with 21 input channels (7 consecutive frames, each with 3 color channels). When the frame of interest was within the first three or last three frames of the pullback, black dummy frames were inserted to maintain a consistent input size for the model. All data was pseudonymized prior to use.

*Model architecture*

A state-of-the art image segmentation network, no-new-Net (nnU-Net) was adopted for multiclass semantic segmentation.(1) As a self-configuring framework, given a dataset and a task, the nnU-Net automatically defines model architecture, training and inference pipelines. For the present task, the encoder-decoder architecture consisted of seven 2D convolutional blocks with skip-connections via feature map concatenation. The number of filters used for the encoding branch was 32, 64, 128, 256, 480, 480, 480. For all convolutional layers, 3 x 3 kernels, leaky ReLu activation functions, and instance normalization were used, resulting in a neural network with 41 million learnable parameters.

*Model training*

The nnU-Net was trained for 1000 epochs, using a batch size of 4, and automatically decreasing the learning rate based on a polynomial function every epoch to improve convergence (Supplementary Method 4). The model was trained using stochastic gradient descent with Nesterov momentum of 0.99 as optimizer with randomly initialized weights. Data augmentation was applied on the fly and included random rotation, scaling, mirroring and gamma correction. A combination of equally weighted cross-entropy and Dice losses was minimized during model training. Deep supervision was applied, injecting gradients deeper into the network during training by adding auxiliary losses to the intermediate decoder layers. Five-fold cross-validation was used on the training set. With this process, the training set was divided into five equal parts or ‘folds’. The model was trained on four of the five folds and evaluated on the fifth. This process was repeated five times so that each fold served four times for training and once for evaluation, effectively resulting in five different models. The final model used during model testing, the predicted segmentation was obtained by ensemble averaging the outputs of the five trained models. Training was conducted in approximately 3 days on a compute cluster with GPUs similar to those used for testing. Parallel processing across the validation folds was utilized to reduce training times compared to sequential execution.

*Post-processing*

Segmentation predictions were post-processed using connected component analysis for 4-connectivity objects for all classes but plaque rupture and thrombus. With 4-connectivity, groups of pixels that share orthogonal edges are considered continuous, while diagonally connected pixels are considered separate. Pixels of areas smaller than applied thresholds were reclassified to the largest neighboring class. Applied thresholds were 0.03 mm^2^ for the classes background and lumen, 0.10 mm^2^ for guidewire, intima and lipid plaques, and 0.01 mm^2^ for calcified plaques, media and side branches.

## Method S4 Mean overall Dice score without postprocessing in early stopping experiment

|  | Validation folds | | | | | |
| --- | --- | --- | --- | --- | --- | --- |
| Epoch | 1 | 2 | 3 | 4 | 5 | Mean |
| Best* | 0.660 | 0.646 | 0.676 | 0.635 | 0.659 | 0.655 |
| 200 | 0.542 | 0.600 | 0.585 | 0.561 | 0.541 | 0.565 |
| 400 | 0.604 | 0.592 | 0.597 | 0.601 | 0.575 | 0.594 |
| 600 | 0.595 | 0.596 | 0.596 | 0.589 | 0.577 | 0.590 |
| 800 | 0.630 | 0.621 | 0.639 | 0.633 | 0.613 | 0.627 |
| 1000 | 0.660 | 0.647 | 0.676 | 0.649 | 0.659 | 0.658 |

* Defined as epoch with the highest pseudo Dice score (i.e. epoch 940, 996, 986, 895, 1000 for each of the 5 folds, respectively). The pseudo Dice approximates the overall performance of the model by evaluating a subset of frames in the validation set.

# Supplementary Results

## Table S1 Class distribution within internal test set

|  | Training frames (n=2808) | Test frames (n=218) |  |
| --- | --- | --- | --- |
| Guidewire artefact | 2808 (100%) | 218 (100%) |  |
| Lumen | 2808 (100%) | 218 (100%) |  |
| Side branch | 485 (17.3%) | 44 (20.2%) |  |
| Intima | 2808 (100%) | 218 (100%) |  |
| Media | 2668 (95.0%) | 206 (94.5%) |  |
| Lipid | 1509 (53.7%) | 102 (46.8%) |  |
| Calcium | 949 (33.8%) | 53 (24.3%) |  |
| Thrombus | 263 (9.4%) | 21 (9.6%) |  |
| Plaque rupture | 191 (6.8%) | 6 (2.8%) |  |

## Table S2 Confusion matrix for frame-wise lipid plaque identification

|  |  | **Reference standard** | | |
| --- | --- | --- | --- | --- |
|  |  | Present | Absent | Total |
| **Prediction** | Predicted | 100 | 18 | 118 |
|  | Not predicted | 2 | 98 | 100 |
|  | Total | 102 | 116 | 218 |

## Table S3 Confusion matrix for frame-wise lipid-rich plaque identification

|  |  | **Reference standard** | | |
| --- | --- | --- | --- | --- |
|  |  | Present | Absent | Total |
| **Prediction** | Predicted | 73 | 13 | 86 |
|  | Not predicted | 3 | 129 | 132 |
|  | Total | 76 | 142 | 218 |

## Table S4 Confusion matrix for frame-wise calcified plaque identification

|  |  | **Reference standard** | | |
| --- | --- | --- | --- | --- |
|  |  | Present | Absent | Total |
| **Prediction** | Predicted | 47 | 11 | 58 |
|  | Not predicted | 6 | 154 | 160 |
|  | Total | 53 | 165 | 218 |

## Table S5 Confusion matrix for frame-wise side branch identification

|  |  | **Reference standard** | | |
| --- | --- | --- | --- | --- |
|  |  | Present | Absent | Total |
| **Prediction** | Predicted | 36 | 9 | 45 |
|  | Not predicted | 10 | 163 | 173 |
|  | Total | 46 | 172 | 218 |

## Table S6 Confusion matrix for frame-wise thrombus identification

|  |  | **Reference standard** | | |
| --- | --- | --- | --- | --- |
|  |  | Present | Absent | Total |
| **Prediction** | Predicted | 19 | 3 | 22 |
|  | Not predicted | 2 | 194 | 196 |
|  | Total | 21 | 197 | 218 |

## Table S7 Confusion matrix for frame-wise plaque rupture identification

|  |  | **Reference standard** | | |
| --- | --- | --- | --- | --- |
|  |  | Present | Absent | Total |
| **Prediction** | Predicted | 5 | 9 | 14 |
|  | Not predicted | 1 | 203 | 204 |
|  | Total | 6 | 212 | 218 |

## Figure S1 Correlation and bias in lipid plaque quantification in the internal test set


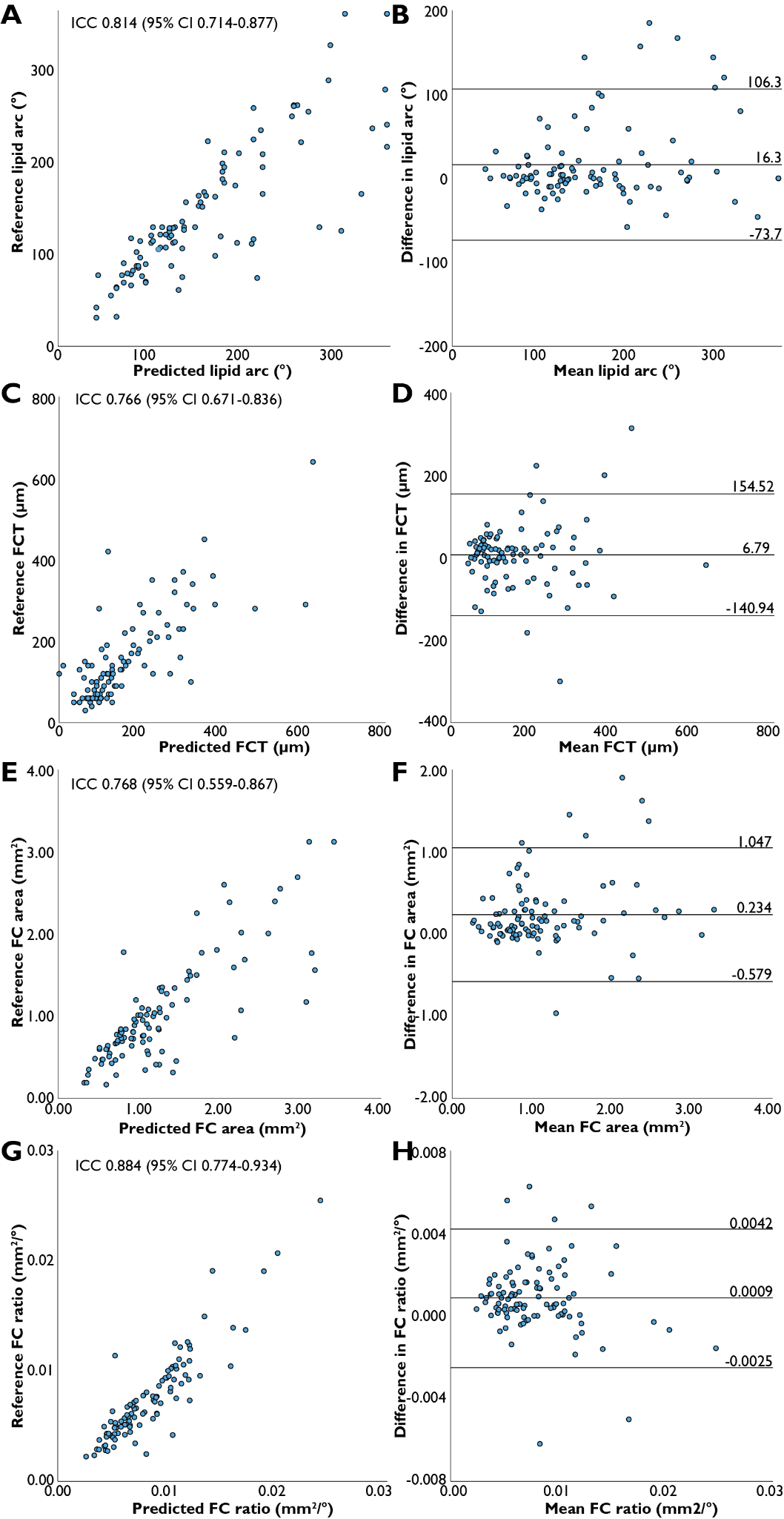


Scatterplots and Bland-Altman plots for lipid arc (A-B), minimum fibrous cap thickness (C-D), fibrous cap area (E-F) and FC ratio (G-H). Differences in quantification values were calculated as predicted value minus reference standard value and values are reported as mean with 95% confidence intervals. A value above zero represents a mean overestimation by the algorithm and a value below zero represents a mean underestimation by the algorithm.

CI confidence interval; FC fibrous cap; minFCT minimum fibrous cap thickness; ICC intraclass correlation.

## Figure S2 Correlation and bias in calcified plaque quantification in the internal test set


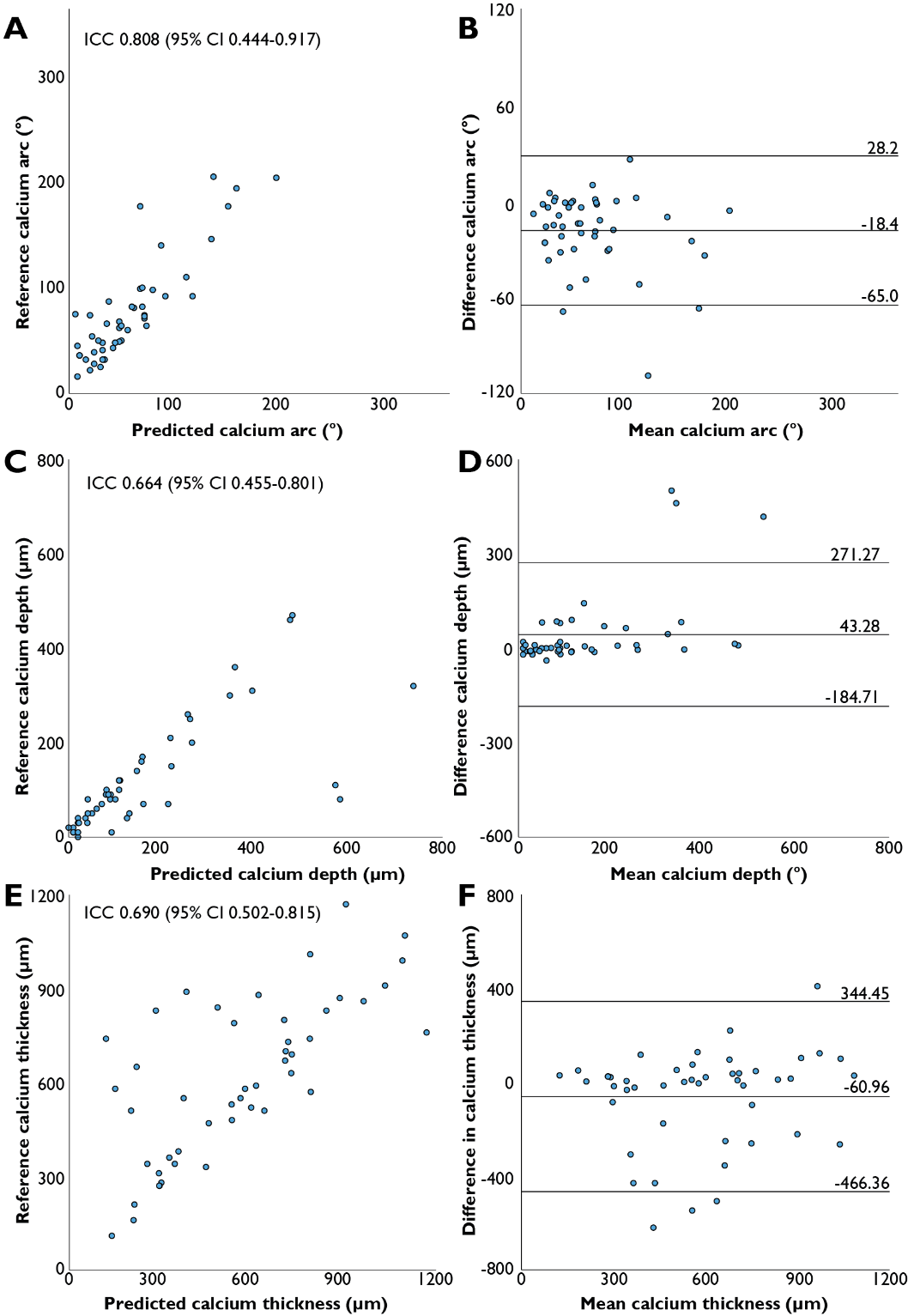


Scatterplots and Bland-Altman plots for calcium arc (A-B), calcium depth (C-D) and calcium thickness (E-F). Differences in quantification values were calculated as predicted value minus reference standard value and values are reported as mean with 95% confidence intervals. A value above zero represents a mean overestimation by the algorithm and a value below zero represents a mean underestimation by the algorithm.

CI confidence interval; ICC intraclass correlation.

## Figure S3 Bias in plaque quantification in the external test set


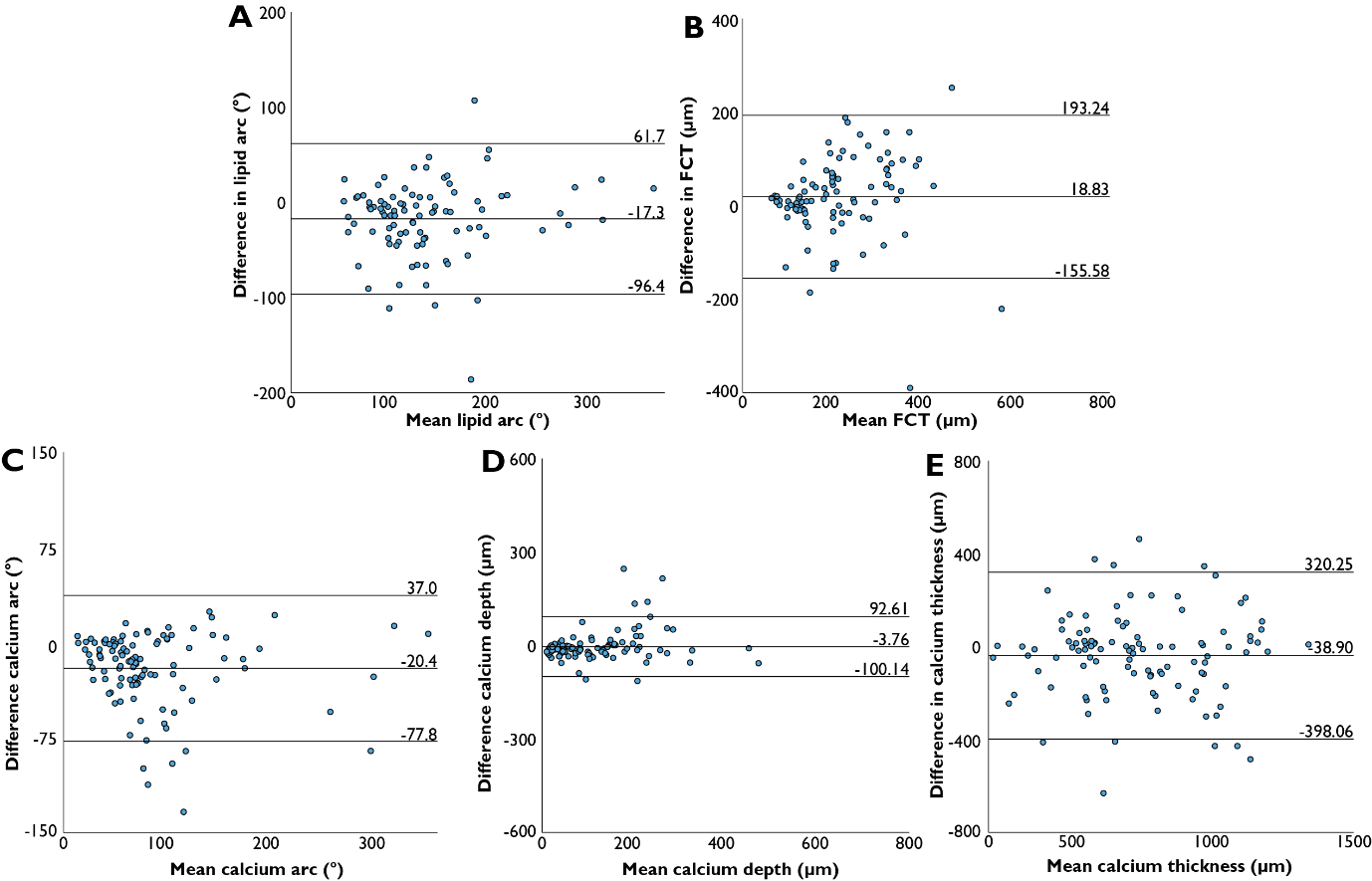


Bland-Altman plots for lipid arc (A), minimum fibrous cap thickness (B), calcium arc (C), calcium depth (D) and calcium thickness (E). Differences in quantification values were calculated as predicted value minus reference standard value and values are reported as mean with 95% confidence intervals. A value above zero represents a mean overestimation by the algorithm and a value below zero represents a mean underestimation by the algorithm.

FCT fibrous cap thickness.

# Supplementary References

1. Isensee F, Jaeger PF, Kohl SAA, Petersen J, Maier-Hein KH. nnU-Net: a self-configuring method for deep learning-based biomedical image segmentation. Nat Methods. 2021;18(2):203-11.
